# Young Woman with Unexplained Neutropenia and Neutrophils with Bilobed Nuclei: Marrow Findings

**DOI:** 10.1155/2023/8844577

**Published:** 2023-09-25

**Authors:** Martin Barnes, Victoria Shklar, Dipen Patel, Harry Staszewski

**Affiliations:** ^1^Mather Hospital, Northwell Health, Hematology Oncology Fellowship, 75 North Country Rd, Port Jefferson, NY 11777, USA; ^2^New York Cancer and Blood Specialists, 49 Nesconset Highway, Port Jefferson Station, NY 11776, USA

## Abstract

A 27-year-old female with a history of chronic sinusitis was referred for the evaluation of asymptomatic neutropenia. The differential demonstrated a mild neutropenia, which eventually resolved on subsequent evaluation. The liver and the spleen were not palpable. Peripheral flow cytometry was normal. Peripheral blood smear (PBS) demonstrated numerous Pelger–Huet anomalous neutrophils with characteristic “pince-nez” nuclei, without significant abnormalities in the other cell lines. Due to the benign clinical nature of hereditary PHA, a bone marrow biopsy is almost never required. However, our patient's persistent and worsening neutropenia was unusual for hereditary PHA, so a bone marrow biopsy was performed to rule out MDS and leukemia. Our patient's bone marrow smears showed dysplastic changes to other cell lines including the megakaryocytes and erythroid precursors. Due to our patient's young age and concern that she may have a more serious marrow disorder, genetic testing was pursued. Germline testing in the LBR gene revealed a heterozygous pathogenic mutation, namely, the PR57837.17 variant, confirming the diagnosis of hereditary disease. The bone marrow biopsy performed on our patient illustrates that the presence of dysplasia does not rule out hereditary PHA and further genetic testing should be done in the appropriate clinical scenario. This case was an atypical presentation of hereditary PHA with confounding morphological features that would typically classify the disease as an acquired or pseudo-PHA, hence acting as a Pseudo-Pseudo-Pelger–Huet Anomaly.

## 1. Introduction

Among the different lines of white blood cells (WBC) present in normal human blood, the neutrophil or polymorphonuclear leukocyte (PMN) is the most common cell type. It has a unique morphology compared to other leukocytes due to its multilobulated or segmented nucleus, which allows for rapid travel through blood vessels via diapedesis. Typical neutrophil nuclei contain 3 or 4 lobes separated by thin filaments [1]. Many diseases are known to alter neutrophil anatomy. Some lead to hyperlobulation, such as hypovitaminosis of B12 or folate, along with a variety of other causes including both acquired and inherited diseases [2]. In contrast, other diseases lead to hypolobulation of the nuclei, typically presenting with 2 lobes. One of the most established variants of a hypolobulated neutrophil is the Pelger–Huet anomaly (PHA). This anomaly can be inherited or acquired, and we will discuss further the clinicopathological implications of both scenarios. We report a case of inherited PHA and atypical bone marrow biopsy findings in a young woman with neutropenia and dysplasia in multiple cell lines.

## 2. Case Presentation

A 27-year-old female with a history of chronic sinusitis was referred for the evaluation of asymptomatic neutropenia. She had no history of smoking, illicit drug, or alcohol use. There was no history of viral infections, including Epstein–Barr virus, hepatitis, or human immunodeficiency virus. She was not experiencing any weight loss, chest pain, or shortness of breath. She denied any recent travel. There was no family history of a blood disorder. She had never received any blood transfusions and felt well overall. Her only medications were fluticasone propionate and an oral contraceptive pill, which she had recently discontinued after taking for 7 years. Complete blood count obtained as part of her routine annual physical by her primary care provider revealed mild leukopenia with a white blood cell (WBC) count of 3300/*μ*L (normal 4000−10,500/*μ*L) and an absolute neutrophil count (ANC) of 1500/*μ*L (normal 1500–6500/*μ*L); other cell lines were within normal limits. The review of outpatient testing from the prior four years demonstrated a persistently normal WBC count. On initial evaluation in our office, her WBC count was slightly decreased at 3600/*μ*L, hemoglobin was 15.7 g/dL (normal 13−17 g/dL), and platelet count was 219,000/*μ*L (normal 150,000–500,000/*μ*L). The differential demonstrated a mild neutropenia with an ANC of 1100/*μ*L, which eventually resolved on subsequent evaluation. The liver and the spleen were not palpable. Peripheral flow cytometry was normal. Peripheral blood smear (PBS) demonstrated numerous Pelger–Huet anomalous neutrophils with characteristic “pince-nez” nuclei, without significant abnormalities in the other cell lines.

Due to the benign clinical nature of hereditary PHA, a bone marrow biopsy is almost never required. However, our patient's persistent and worsening neutropenia is unusual for hereditary PHA; so, a bone marrow biopsy was performed to rule out MDS and leukemia as a possible cause of her presumed pseudo-PHA.

Our patient's bone marrow smears showed dysplastic changes to other cell lines including the megakaryocytes and erythroid precursors. Due to our patient's young age and concern that she may have a more serious marrow disorder, genetic testing was pursued. Germline testing was performed on a single gene, the LBR gene, and revealed a heterozygous pathogenic mutation, namely, the PR57837.17 variant, confirming the diagnosis of hereditary disease. The medical genetics company Invitae processed the genomic DNA obtained from the submitted sample. The sample was enriched for targeted regions using a hybridization-based protocol and sequenced using Illumina technology. The chromosomal coordinate was LBR c.1741_1742del (p.Val581 Profs*∗*6). Gene: LBR, transcript: NM_002296.3. The variant allele fraction is not given as per the Invitae report. This variant is quite rare and is classified as pathogenic. Genetic counseling and testing family were recommended and planned; however, the relatives were unavailable for testing in this case. The mutation is very rare, and the odds of both parents having a mutation are low and unlikely to result in significant health effects in heterozygous offspring.

## 3. Discussion

The incidence of hereditary PHA has been estimated around 1 in 6000 with a codominant inheritance pattern [3]. Heterozygous patients typically remain asymptomatic, and outside of one study have not been shown to have significant differences in neutrophil cytoplasmic granular enzymes, superoxide production, or chemotaxis. However, patients with homozygous disease can present with a range of complications from macrocephalus, ventricular septal defects, psychomotor retardation, and skeletal complications, including polydactyl, short metacarpals, disproportionate body stature, and a constellation of hydrops fetalis, ectopic calcifications, and “moth-eaten” skeletal dysplasia (HEM syndrome or Greenberg dysplasia). Homozygotes can also experience abnormal dentition, grand mal seizures, and eczema [1].

One paper compared neutrophil morphology between hereditary PHA and pseudo-PHA and found that neutrophils in MDS were similar to those of hereditary PHA, but neutrophils of AML showed more heterogeneous pattern in lobulation and more chromatin hypercondensation. In general, this paper noted that pseudo-PHA anomalies are more heterogeneous in morphology compared to the familial anomaly [4].

It is important to remember that several etiologies can lead to an acquired PHA including medications such as valproic acid, docetaxel, and chemical exposures like benzenes, and hematologic diseases such as MDS, leukemia, and Fanconi anemia [5, 6]. Sometimes, infectious disease states can also lead to these changes as can be seen in cases of malaria, influenza, and lupus and more recently has been seen in SARS-COV-2 infections [7]. Acquired PHA has also been linked to the use of tacrolimus in organ transplant recipients with an incidence around 5.3% in one series of kidney transplant patients [8, 9].

To distinguish hereditary PHA from acquired or pseudo-PHA, a bone marrow biopsy may be performed to rule out conditions associated with acquired PHA. One study showed that the presence of 2 or more of the following findings is typically suggestive of an acquired PHA: hypercellularity, morphologic dysplasia, clonal cytogenetic abnormalities, and increased blasts. The degree of heterogeneity may also be indicative of origin, as patients with myelodysplastic syndrome (MDS) or acute myeloid leukemia demonstrate a more diverse range of PHA morphologies as compared to a more uniform display seen in hereditary PHA.

**Figure 1:**
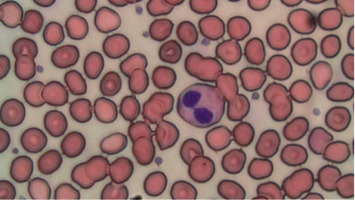
Peripheral blood smear showing classic “pince-nez” findings consistent with the Pelger–Huet anomaly (PHA) (Wright-Giemsa stain × 100).

**Figure 2:**
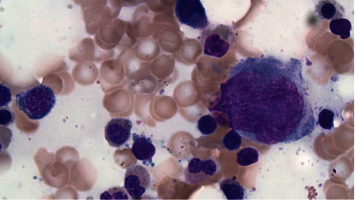
Bone marrow biopsy showing evidence of dysmegakaryopoiesis (Giemsa stain × 100).

**Figure 3:**
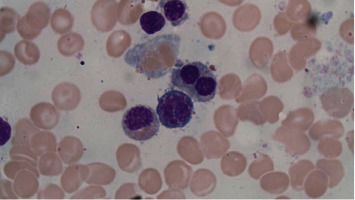
Bone marrow biopsy showing evidence of dyserythropoiesis (Giemsa stain × 100).

While flow cytometry and cytogenetics from our patient's bone marrow aspirate were normal, additional morphology findings were as follows: Figure 1 displays classic findings of the “pince-nez” findings, which led to the suspicion of underlying PHA. Her bone marrow smears, as displayed in Figures 2 and 3, show dysplastic changes to other cell lines including the megakaryocytes and erythroid precursors. When dysplastic changes are seen, further testing is rarely pursued as the diagnosis of pseudo-PHA is made and attributed to MDS. However, due to our patient's young age and concern that she may have a more serious marrow disorder, genetic testing was pursued. Germline testing in the LBR gene revealed a heterozygous pathogenic mutation, namely, the PR57837.17 variant, confirming the diagnosis of hereditary disease.

To our knowledge, the bone marrow findings in true hereditary PHA are not well described except for one paper from the Japanese literature [4]. This paper noted that the lack of lamin B receptor protein in hereditary PHA results in hypolobulation and chromatin hypercondensation in neutrophils. They also found that one third of megakaryocytes were binucleated like the PHA neutrophils. However, for other cells, such as erythroblasts and lymphocytes, only chromatin hypercondensation was observed [5].

The bone marrow biopsy performed on our patient illustrates that the presence of dysplasia does not rule out hereditary PHA and further genetic testing should be done in the appropriate clinical scenario. This case was an atypical presentation of hereditary PHA with confounding morphological features that would typically classify the disease as an acquired or pseudo-PHA, hence acting as a Pseudo-Pseudo-Pelger–Huet Anomaly.

Patients with hereditary PHA do not typically suffer from cytopenias. In our patient, the presence of absolute neutropenia and the peripheral blood presence of Pelger–Huet neutrophils raised the suspicion of possible MDS. Since there are few descriptions of the bone marrow in patients with hereditary PHA in the literature, our case demonstrates that there may be some overlap in the appearance of the bone marrow between hereditary PHA and pseudo-PHA. This case is a reminder that unrelated cytopenias can occur in patients with hereditary PHA and molecular studies may be helpful in clarifying the diagnosis.

## Data Availability

No data were used to support this study.
